# Supplementary material for: Development and evaluation of inhalable composite niclosamide-lysozyme particles: A broad-spectrum, patient-adaptable treatment for coronavirus infections and sequalae
Source: PLoS One. 2021 Feb 11;16(2):e0246803. doi: 10.1371/journal.pone.0246803 (PMC7877651; doi:10.1371/journal.pone.0246803)
Supplement: S1 Table — (DOCX) [file pone.0246803.s001.docx]

**S1 Table. D-optimal subset utilized for constrained mixtures DoE**

| **Run** | **X1 (NIC-M)** | **X2 (hLYS)** | **X3 (sucrose)** | **X4 (polysorbate 80)** | **Dimension** |
| --- | --- | --- | --- | --- | --- |
| 1 | 0.0030 | 0.6000 | 0.3965 | 0.0005 | 0 |
| 2 | 0.0100 | 0.6000 | 0.3895 | 0.0005 | 0 |
| 3 | 0.0030 | 0.7965 | 0.2000 | 0.0005 | 0 |
| 4 | 0.0100 | 0.7895 | 0.2000 | 0.0005 | 0 |
| 5 | 0.0030 | 0.6000 | 0.3950 | 0.0020 | 0 |
| 6 | 0.0030 | 0.7950 | 0.2000 | 0.0020 | 0 |
| 7 | 0.0100 | 0.6000 | 0.3880 | 0.0020 | 0 |
| 8 | 0.0100 | 0.7880 | 0.2000 | 0.0020 | 0 |
| 14 | 0.0100 | 0.7888 | 0.2000 | 0.0012 | 1 |
| 21 | 0.0030 | 0.6979 | 0.2979 | 0.0012 | 2 |
| 23 | 0.0065 | 0.6000 | 0.3922 | 0.0012 | 2 |
| 25 | 0.0065 | 0.6965 | 0.2965 | 0.0005 | 2 |
