# Supplementary material for: Development and evaluation of inhalable composite niclosamide-lysozyme particles: A broad-spectrum, patient-adaptable treatment for coronavirus infections and sequalae
Source: PLoS One. 2021 Feb 11;16(2):e0246803. doi: 10.1371/journal.pone.0246803 (PMC7877651; doi:10.1371/journal.pone.0246803)
Supplement: S2 Table — (DOCX) [file pone.0246803.s002.docx]

**S2 Table. Primer sequences used for the quantification of viral particles**

| **Primer name** | **Sequence** |
| --- | --- |
| MERS N3 Forward | GGG TGT ACC TCT TAA TGC CAA TTC |
| MERS N3 Reverse | TCT GTC CTG TCT CCG CCA AT |
| MERS N3 probe | 5’FAM-ACC CCT GCG CAA AAT CGT-BHQ1 3’ |
| SARS-CoV-2 Forward | CAC ATT GGC ACC CGC AAT C |
| SARS-CoV-2 Reverse | GAG GAA CGA GAA GAG GCT TG |
| SARS-CoV-2 Probe | 5’FAM-ACT TCC TCA AGG AAC AAC ATT GCC A-BHQ1 3’ |
