# Supplementary material for: Development and evaluation of inhalable composite niclosamide-lysozyme particles: A broad-spectrum, patient-adaptable treatment for coronavirus infections and sequalae
Source: PLoS One. 2021 Feb 11;16(2):e0246803. doi: 10.1371/journal.pone.0246803 (PMC7877651; doi:10.1371/journal.pone.0246803)
Supplement: S3 Table — (DOCX) [file pone.0246803.s003.docx]

**S3 Table: Spray pattern analysis of varying concentrations of NIC-hLYS emitted from the VP7 Aptar® nasal spray device**

|  | | **Spray Pattern 2 cm** | | | **Spray Pattern 5 cm** | | |
| --- | --- | --- | --- | --- | --- | --- | --- |
| **Concentration (mg/mL)** | **Plume Angle (^o^)** | **Spray Area (mm^2^)** | **Max Diameter**  **(mm)** | **Min Diameter**  **(mm)** | **Spray Area**  **(mm^2^)** | **Max Diameter**  **(mm)** | **Min Diameter**  **(mm)** |
| 10 | 42.3 ± 0.8 | 432 ± 5.0 | 24.4 ± 0.2 | 23.4 ± 0.1 | 1398 ± 71.4 | 49.6 ± 1.1 | 38.0 ± 1.4 |
| 25 | 45.5 ± 2.7 | 434 ± 5.7 | 26.7 ± 1.1 | 22.9 ± 0.1 | 1799 ± 87.5 | 58.8 ± 0.9 | 43.6 ± 1.0 |
| 50 | 58.6 ± 1.6 | 470 ± 2.2 | 28.5 ± 0.3 | 23.4 ± 0.2 | 2668 ± 591.8 | 64.0 ± 8.1 | 55.1 ± 5.6 |
