# Supplementary material for: Development and evaluation of inhalable composite niclosamide-lysozyme particles: A broad-spectrum, patient-adaptable treatment for coronavirus infections and sequalae
Source: PLoS One. 2021 Feb 11;16(2):e0246803. doi: 10.1371/journal.pone.0246803 (PMC7877651; doi:10.1371/journal.pone.0246803)
Supplement: S4 Table — (DOCX) [file pone.0246803.s004.docx]

**S4 Table: Osmolality of varying concentrations of NIC-hLYS reconstituted in 0.45% sodium chloride**

| **Concentration (mg/mL)** | **Osmolality (mOsmol/kg)** |
| --- | --- |
| 10 | 156 ± 2 |
| 25 | 179 ± 2 |
| 50 | 208 ± 1 |
