# Supplementary material for: Development and evaluation of inhalable composite niclosamide-lysozyme particles: A broad-spectrum, patient-adaptable treatment for coronavirus infections and sequalae
Source: PLoS One. 2021 Feb 11;16(2):e0246803. doi: 10.1371/journal.pone.0246803 (PMC7877651; doi:10.1371/journal.pone.0246803)
Supplement: S1 Fig — Spray drying (B) did not result in additional formation of soluble aggregates compared to the unprocessed control (A). NIC-hLYS reconstituted at a concentration of 25 mg/mL (C), 50 mg/mL (D), 75 mg/mL (E), and 100 mg/mL (F) was nebulized over the course of 2 minutes from an Aerogen Solo® vibrating mesh nebulizer. This process did not result in additional aggregation compared to the unprocessed control. (DOCX) [file pone.0246803.s005.docx]

**
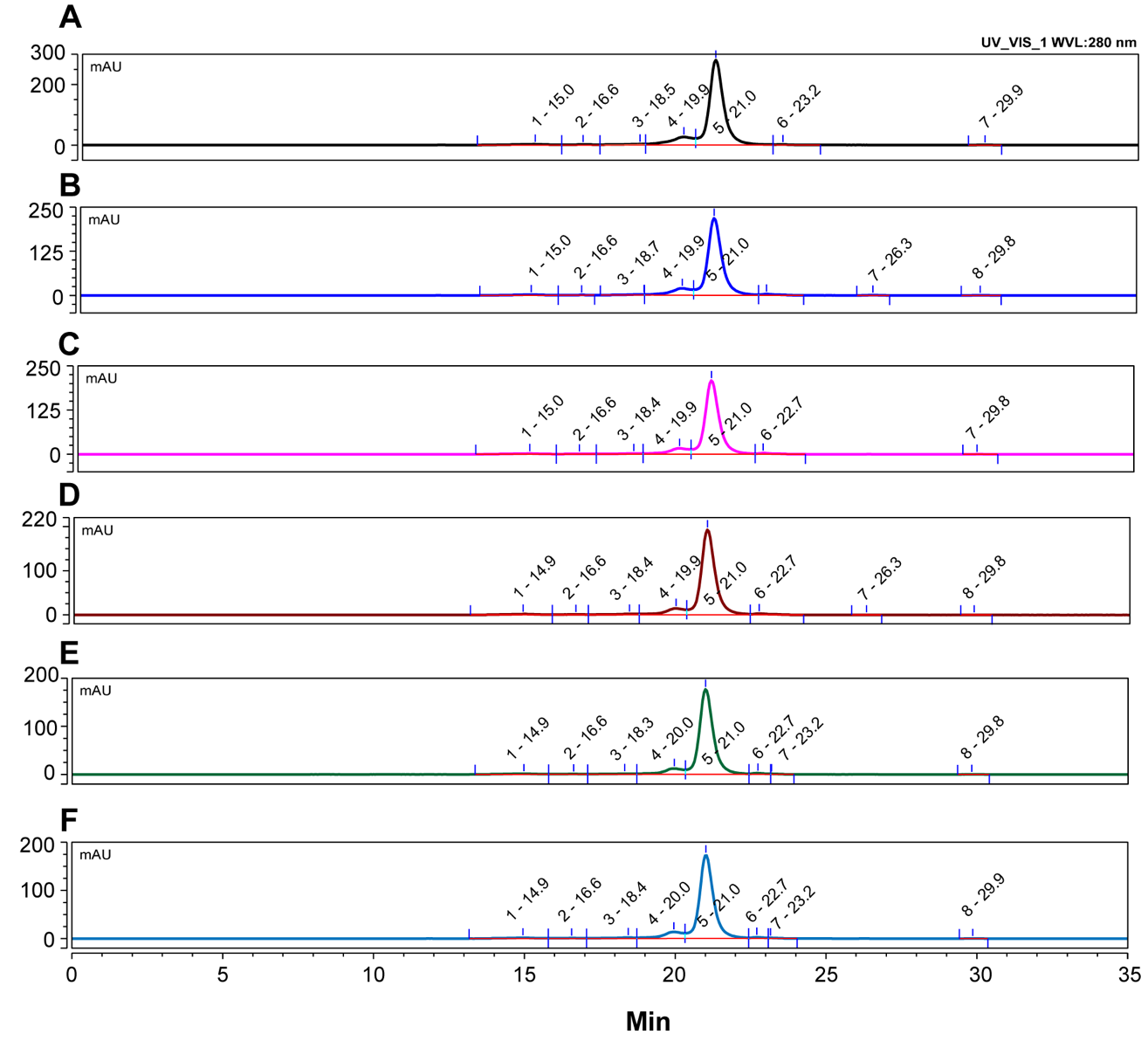
**

**S1 Fig. Effect of processing and nebulization on hLYS aggregation.** Spray drying (B) did not result in additional formation of soluble aggregates compared to the unprocessed control (A). NIC-hLYS reconstituted at a concentration of 25 mg/mL (C), 50 mg/mL (D), 75 mg/mL (E), and 100 mg/mL (F) was nebulized over the course of 2 minutes from an Aerogen Solo® vibrating mesh nebulizer. This process did not result in additional aggregation compared to the unprocessed control.
